# Infantile‐onset Pompe disease with neutropenia: Treatment decisions in the face of a unique phenotype

**DOI:** 10.1002/jmd2.12337

**Published:** 2022-09-27

**Authors:** Mary Riedy, Jeff F. Zhang, Taosheng Huang, Anil Kumar Swayampakula

**Affiliations:** ^1^ Department of Pharmacy Practice, School of Pharmacy and Pharmaceutical Sciences University at Buffalo Buffalo New York USA; ^2^ Jacobs School of Medicine and Biomedical Sciences University at Buffalo Buffalo New York USA; ^3^ Division of Genetics, Department of Pediatrics University at Buffalo Buffalo New York USA; ^4^ Division of Critical Care Medicine, Department of Pediatrics, John R. Oishei Children's Hospital University at Buffalo Buffalo New York USA

**Keywords:** atrial flutter, enzyme replacement therapy, *GAA* gene, infantile‐onset Pompe disease (IOPD), neutropenia, ventricular hypertrophy

## Abstract

Infantile‐onset Pompe disease manifests with early signs of cardiomyopathy during the first few days to weeks of life. We present the case of a newborn born via emergency cesarean section with atrial flutter and moderate biventricular hypertrophy who was diagnosed with Pompe disease on New York State newborn screen. Diagnosis was confirmed with repeat leukocyte acid alpha‐glucosidase (GAA) enzyme activity, *GAA* gene sequencing, urine Hex4, and evaluation of Cross‐Reactive Immunological Material (CRIM) status. The patient was also found to be persistently neutropenic which to our knowledge has not been previously reported in the literature in association with Pompe disease. This report highlights the impact that newborn screening had on time to diagnosis and initiation of treatment with enzyme replacement therapy. We also discuss how our patient's concurrent neutropenia impacted decision making related to immune tolerance induction prior to starting enzyme replacement therapy.

**SYNOPSIS**

This case highlights the importance of newborn screening and the impact of neutropenia on treatment decisions in a patient with infantile‐onset Pompe disease.

## 1 INTRODUCTION

Pompe disease, also known as glycogen storage disease type II, is caused by a deficiency in functional lysosomal acid alpha‐glucosidase (GAA) enzyme. This enzyme is responsible for the breakdown of glycogen to glucose within the lysosomes. Thus, systemic accumulation of glycogen in various body tissues causes damage to skeletal and smooth muscle as well as cardiac and hepatic dysfunction.^1^ Pompe disease is inherited in an autosomal recessive manner and is caused by biallelic pathogenic variants in the *GAA* gene. Patients with Pompe disease can be classified as infantile‐onset Pompe disease (IOPD) or late‐onset Pompe disease (LOPD). The distinguishing feature for this classification is not the specific age at onset of symptoms, but rather the presence of cardiomyopathy during the newborn period in patients with IOPD.^1^

While the incidence of Pompe disease has been estimated to be 1:40000,^2^ IOPD, characterized by cardiomyopathy or cardiac involvement before 12 months of age, presents in approximately 1:100000–1:200000 live births.^3^ In a global study of 168 cases of IOPD, the mean age of presentation was 2.7 ± 2.5 months, with hypotonia (82%) and heart failure (62%) being the most common presenting symptoms.^3^ Deposition of glycogen within cardiac muscle causes disruption of cardiomyocyte conduction circuits, resulting in the development of various types of arrhythmia, including supraventricular tachycardia and Wolff‐Parkinson‐White syndrome.^4^

In most US states, Pompe disease has been added to the newborn screening panel and early detection can assist providers in diagnosis and prompt initiation of enzyme replacement therapy (ERT). ERT with alglucosidase alfa (Lumizyme®) was FDA approved in 2006 for the treatment of IOPD and LOPD.^5^ Alglucosidase alfa provides patients with supplemental enzyme to assist in the breakdown of glycogen in lysosomes. Recently published case reports indicate that early treatment leads to better outcomes, especially for patients with IOPD.^6^, ^7^, ^8^, ^9^, ^10^ We present a case of IOPD who presented with atrial flutter in the immediate newborn period along with neutropenia, for which treatment decisions had to be amended for this unique phenotype.

## 2 CASE REPORT

Our patient is a male infant born at 37 6/7 weeks gestation, large for gestational age (LGA) (birth weight 4.18 kg), via cesarean section to a primigravid 29‐year‐old mother with pertinent maternal history of obesity and gestational diabetes. On the morning of patient delivery, the mother had presented for a routine prenatal care appointment when the fetal heart rate was noted to be over 200 beats per minute (bpm) without hydropic features. A fetal echocardiogram was performed which revealed atrial flutter, prompting an emergent caesarian section. The patient's Apgar scores were recorded as 8 and 9 at 1 and 5 min, respectively. Following delivery, the patient was cyanotic, with persistent tachycardia (heart rate ~230 bpm) consistent with atrial flutter, required cardioversion and was monitored in the neonatal intensive care unit (ICU). Chest x‐ray was unremarkable, and echocardiogram (ECHO) demonstrated moderate biventricular hypertrophy without outflow obstruction. Based on ECHO findings and maternal history, it was suspected that uncontrolled gestational diabetes may have been a causative factor of the patient's cardiomyopathy.

On day of life (DOL) #7, New York State newborn screening results revealed a reduction in GAA enzyme activity. Second tier DNA sequencing confirmed the diagnosis of Pompe disease with two pathogenic variants, p.Met318Thr (c.953T>C) and p.Asn570Lys (c.1710C>G) reported in the *GAA* gene. Familial testing in parental samples confirmed the variants were in trans. Additionally, he had an elevated creatine kinase (CK) level of 1197 unit/L and an absolute neutrophil count (ANC) of 693 cells/μL, decreased from 3996 cells/μL at birth, without signs of infection. The patient was discharged home on DOL #10 with further evaluation as an outpatient by Genetics, Immunology, Hematology, Cardiology, and Infectious Diseases teams.

Further immunology workup to identify potential causes for the patient's persistent neutropenia was indicated. IgA was found to be low at 5.0 mg/dL, IgE elevated at 25 units/ml, and IgG and IgM normal at 564 mg/dL and 31 mg/dL, respectively. Lymphocyte flow cytometry (CD3, CD4, CD8, C19, NK cell markers) was found to be within normal ranges. Dihydrorhodamine assay showed neutrophil fluorescence within normal limits. A Bone Marrow Failure Syndromes Panel and Primary Immunodeficiency panel, analyzing 429 genes (Invitae PATH4ward Program), were both negative. Send‐out laboratory testing related to Pompe disease resulted on DOL #24‐30, including CRIM positive status, elevated urine glucotetrasaccharides (Hex4) at 18.6 mmol/mol Cr, and repeat GAA enzyme, leukocyte of 0.07 nmol/h/mg from blood sample.

The patient was admitted on DOL #37 to the pediatric ICU for peripherally inserted central catheter (PICC) line placement and initiated on alglucosidase alfa infusion at 40 mg/kg/dose on DOL #38. He had been thriving well at home without signs of fever or infection. Prior to any ERT, the patient's ANC was found to be further decreased to 300 cells/μL, with medication‐induced neutropenia or an infectious process determined to be unlikely etiologies. Due to the patient's CRIM positive status and persistent neutropenia, immune tolerance induction (ITI) with methotrexate or other immune modulating therapies was deferred. The patient's first ERT was tolerated with no adverse events, and he was discharged home on prophylactic amoxicillin‐clavulanate daily for severe neutropenia. Initial IgG anti‐drug antibodies were negative.

During the patient's third ERT infusion on DOL #71, he developed a rash around his neck and chin without respiratory distress or abdominal symptoms approximately 3.5 h into the infusion. After administration of intravenous diphenhydramine and stopping the infusion, his symptoms improved and returned to baseline. ERT was reattempted with return of symptoms and perioral pallor; thus, the decision was made to discontinue therapy during that session.

**FIGURE 1:**
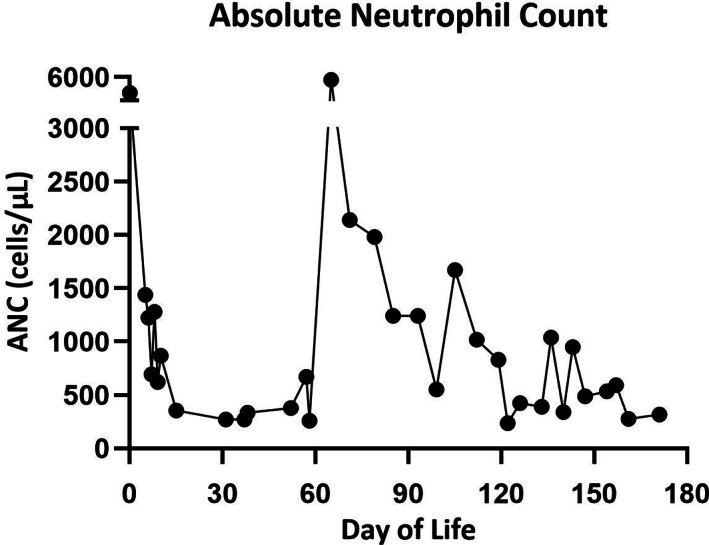
This graph illustrates our patient's absolute neutrophil count over his first 6 months of life^15^

Prior to the fourth ERT infusion on DOL #85, he was premedicated with methylprednisolone, diphenhydramine, and acetaminophen, and the infusion proceeded without any allergic reaction. At this time, the patient's neutropenia was noted to have resolved (ANC = 5670 cells/μL; Figure 1). During the patient's fifth ERT infusion on DOL #99, he developed an anaphylactic reaction with bronchospasm, wheezing, tachypnea, and bradycardia when titrated to 7 mg/kg/h. He responded well to diphenhydramine and methylprednisolone with complete resolution of symptoms. Following this infusion reaction, serum alglucosidase alfa IgE antibody testing was sent and subsequently found to be negative. Serum complement and tryptase were unable to be drawn within the recommended time window due to line access issues. The decision was made to proceed with desensitization protocol for future infusions.

A desensitization protocol was designed and administered successfully with premedication and an alglucosidase alfa dose of 10 mg/kg/dose weekly for three infusions. His initial protocol was a 4‐bag (1:1000, 1:100, 1:10, and 1:1), 16‐step infusion (starting rate: 0.15 μg/kg/h increased 2 to 2.5‐fold every 20 min) administered over approximately 7 h. Once tolerated, we increased to 20 mg/kg/dose and liberalized the initial infusion rate with each infusion. Currently, our patient is tolerating a 6‐step protocol of alglucosidase alfa 20 mg/kg/dose every other week (starting rate: 0.5 mg/kg/h titrating every 20 min as tolerated, not to exceed 5 mg/kg/h).

The patient's ANC has fluctuated over time (Figure 1), despite use of methylprednisolone premedication. At the time of this report, the patient's persistent neutropenia is still due to an unknown cause. At the latest follow‐up at 6 months old, the patient's ANC was 318 cells/μL. Improvement in ventricular hypertrophy was noticed on the most recent ECHO (DOL #149). With regards to alglucosidase alfa IgG anti‐drug antibodies, the patient had tested positive with a titer value of 6400 on DOL #99 and a titer value of 12 800 on DOL #157.

## 3 DISCUSSION

The incidence of atrial flutter in infants under 1 year of age is uncertain due to the rarity of cases. In the largest case study to date, a major hospital in the United States found 52 cases of infantile atrial flutter over a 25 year span, in which 69% of patients presented within the first 48 h of life.^11^ While atrial flutter has previously been reported in chronic Pompe disease patients secondary to degenerative changes caused by dilated cardiomyopathy,^12^ the phenomenon has not been observed acutely in pediatric patients, especially neonates. In a case series of 31 neonates diagnosed with atrial flutter (21/31) and ectopic atrial tachycardia (EAT) (10/31), Pike et al. observed that infants born with atrial flutter or EAT were significantly more likely (*p* = 0.0001) to be born LGA, with maternal diabetes being a common etiology.^13^ In these patients, the pathophysiological mechanism responsible for the disease is thought to be related to macrosomia causing diastolic dysfunction in utero, resulting in stretching of the cardiac atria and disruption of myocardial circuit development.^13^ The incidence of hypertrophic cardiomyopathy ranges from 13% to 44% in infants of diabetic mothers (IDM).^14^ While our patient's arrhythmia and cardiomyopathy seen on ECHO were initially attributed to LGA or IDM status, the results of newborn screening drastically changed treatment decision making for our patient. As of March 2022, only 30 states screen for Pompe disease on newborn screening panels, with New York state having added this screening in 2014. This case highlights the importance of screening and early detection, as a positive newborn screen prompted appropriate additional testing and treatment with enzyme replacement therapy rather than delay of care that could have resulted with continued attribution to LGA or IDM status.

Additionally, while neonatal neutropenia (defined as ANC below 1000 cells/μL) has been estimated to affect approximately 8% of all newborn infants,^15^ the presentation of neonatal neutropenia with IOPD has not previously been reported. Of note, findings of neutropenia have been reported with glycogen storage disease type 1.^16^, ^17^, ^18^ The relationship these two disease processes have with each other is uncertain. Lysosomal α‐1,4‐glucosidase deficiency causes the deposition of glycogen within various tissues of the body, resulting in disruption of extracellular architecture and cellular functioning (including within lymphocytes). However, mature neutrophils produce an isozyme called maltase glucoamylase instead, which has not been observed to be dysfunctional in patients with Pompe disease.^19^ Neonatal neutropenia has been shown to be heavily correlated with increased birth weight^15^ and maternal hypertension,^20^ and it is thought that maternal endothelial dysfunction resulting in placental hypoxia inhibits adequate fetal bone marrow development and myeloid cell production.^21^ Neonatal neutropenia has been observed to resolve spontaneously within 1–9 weeks of birth with continued maturation of the infantile bone marrow postnatally.^22^ However, our patient had recurrent neutropenia which has persisted through 6 months of life (Figure 1).

Once the diagnosis of IOPD was made, education regarding the patient's diagnosis and ERT treatment was provided to the patient's family by a team consisting of a geneticist, a genetic counselor, and a clinical pharmacist. The importance of early initiation of treatment to reduce morbidity and mortality was emphasized to the patient's family. Due to the complexity of the diagnosis, the family sought out a second opinion to aid in their decision‐making process.

Patients classified as CRIM negative have a high propensity to develop high‐sustained antibody titers (HSAT) against alglucosidase alfa therapy due to nearly or totally absent GAA enzyme function, while CRIM positive patients have been proposed to be less likely to develop anti‐drug antibodies due to residual GAA enzyme function.^23^, ^24^ However, prior studies have demonstrated that a small cohort of CRIM positive patients have developed HSAT, defined as ≥51 200 for two or more titers at or beyond a 6 month period.^23^, ^25^ It is unclear why a subset of CRIM positive patients go on to develop HSAT. Various studies have evaluated correlations between genotype and antibody development in CRIM positive patients.^26^, ^27^, ^28^ Bali et al. evaluated 140 patients with 41 gene mutations and suggested missense mutations and in‐frame deletions were likely to result in CRIM positive status, however their study recommends laboratory confirmation.^26^ Desai et al. assessed 25 CRIM positive patients treated without ITI and evaluated anti‐drug antibody development. They concluded that genotyping alone cannot predict anti‐drug antibody development or benefit from ITI.^28^ While standard of care in the treatment of CRIM negative patients is to initiate ITI with rituximab, immunoglobulin, and methotrexate, CRIM positive patients may benefit from low dose methotrexate.^29^ Kazi et al. demonstrated reductions in both sustained anti‐drug antibody titers and incidence of HSAT in IOPD patients prescribed low‐dose methotrexate with the first three ERT cycles compared to a historical cohort without methotrexate.^30^

The initiation of ERT in an IOPD patient with profound and persistent neutropenia of unknown origin presented our team with a unique challenge not previously described in the medical literature. While awaiting final determination of our patient's neutropenia work‐up and confirmation of CRIM status, our team evaluated ITI strategies reported in the literature. Medications utilized include immune modulation therapies not well studied in patients with persistent neutropenia. Our team decided that despite these approaches described in the literature, we would defer ITI and begin ERT. However, our patient have developed IgG anti‐drug antibodies, presently in the low‐titer range. We are continuing to monitor for IgG anti‐drug antibody development monthly while exploring strategies to understand the etiology of the patient's neutropenia.

## 4 CONCLUSION

Although a spectrum of cardiac manifestations have been seen in patients with IOPD, our patient uniquely presented with atrial flutter requiring cardioversion at delivery. Concomitant neutropenia continues to pose a challenge for our patient's case as low ANC is not typically seen in Pompe disease and precluded use of methotrexate for ITI in our CRIM positive IOPD patient. Although our patient was found to have developed anti‐drug antibodies, treatment efficacy has been demonstrated through improvements seen on ECHO and stability in motor function at this time. We highlight a case that emphasizes the importance of including Pompe disease in newborn screening panels and the challenge of managing the development of anti‐drug antibodies to ERT in a patient with IOPD and neutropenia.

## AUTHOR CONTRIBUTIONS

Mary Riedy performed literature review of existing evidence related to this case, outlined the content related to the case report and information, collected and reviewed information related to the patient case, and drafted the manuscript. Jeff F. Zhang performed literature review of related to this case, collected and reviewed information related to the patient case, and drafted the manuscript. Taosheng Huang contributed to review and critical analysis of content related to patient case and manuscript and provided genetic medical expertise/thought generation. Anil Kumar Swayampakula contributed to review and critical analysis of content related to patient case and manuscript and provided critical care medicine expertise/thought generation.

## FUNDING INFORMATION

No funding was utilized for this patient case report.

## CONFLICT OF INTEREST

The authors declare they do not have any conflicts of interest to disclose related to the content of this case report.

## ETHICS STATEMENT

The authors are accountable for all aspects of the accuracy and integrity of this report. As per University at Buffalo policy, this case report does not require IRB review and approval.

## PATIENT CONSENT

Informed consent was obtained from the patient's parent for publication of this patient case report.

## ANIMAL RIGHTS

This article does not contain any studies with animal subjects performed by any of the authors.

## Data Availability

Data related to the patient case was not archived but can be made available upon request.
